# Strong Foundations, Fragile Structures: An Organisational Paradox in the Nursing Practice Environment—A Multicentre Cross-Sectional Study of Portuguese Nurses

**DOI:** 10.3390/healthcare14213808

**Published:** 2026-11-07

**Authors:** Telma Quaresma, Maria Céu Marques, Liliana Mota

**Affiliations:** 1 CHRC—Comprehensive Health Research Centre, University of Évora, 7004-516 Évora, Portugal; telma.quaresma@ulsalg.min-saude.pt (T.Q.); mcmarques@uevora.pt (M.C.M.); 2 Algarve Local Health Unit—Hospital Unit of Portimão, Department of Education, Innovation and Research, 8500-338 Portimão, Portugal; 3 São João de Deus School of Nursing, University of Évora, 7004-516 Évora, Portugal; 4 Portuguese Red Cross Northern Health School, 3720-126 Oliveira de Azeméis, Portugal; 5 RISE-Health, Faculty of Medicine, University of Porto, 4200-319 Porto, Portugal

**Keywords:** nursing practice environment, nursing workforce, organisational factors, nursing leadership, professional practice

## Abstract

**Highlights:**

**What are the main findings?**

- More than one-third of nurses (36.6%) were assigned to an exploratory profile characterised by relatively favourable nursing foundations for quality of care and collegial nurse–physician relations, alongside markedly weaker staffing and resource adequacy (strong foundations/staffing vulnerability profile). Because cluster separation was weak and reproducibility limited, this classification should be regarded as tentative.
- Organisation-level clustering accounted for 4.2–12.8% of the variance in practice-environment dimensions and altered the interpretation of public–private sector differences; only the sector association with nurse participation in Organisational Affairs remained statistically significant across all sensitivity analyses performed.

**What are the implications of the main findings?**

- Aggregate PES-NWI scores and sector-based comparisons may conceal meaningful combinations of organisational strengths and vulnerabilities; multidimensional, organisation-sensitive assessment may better support targeted improvement.
- Nurse participation in organisational affairs emerged as a potentially modifiable organisational characteristic, supporting future longitudinal and intervention studies focused on participatory governance and practice-environment improvement.

**Abstract:**

Background: Aggregate assessments of nursing practice environments may obscure different combinations of organisational strengths and vulnerabilities. Objective: To characterise nurses’ perceptions of practice environments, identify exploratory multidimensional profiles, and examine associated professional and organisational factors. Methods: A multicentre cross-sectional study included 1004 nurses from 29 Portuguese healthcare organisations. Practice environments were assessed using the Practice Environment Scale of the Nursing Work Index. Analyses included hierarchical regression, exploratory cluster analysis, multilevel models, and indirect-effect analyses. Results: Nursing Foundations for Quality of Care (M = 2.78) and Collegial Nurse–Physician Relations (M = 2.70) received the highest ratings, whereas Nurse Participation in Organisational Affairs (M = 2.27) and Staffing and Resource Adequacy (M = 2.28) received the lowest. Three exploratory profiles were identified; 36.6% of nurses (bootstrap 95% interval: 25.5–40.2%) were assigned to a strong foundations/staffing vulnerability profile spanning different favourability classifications, although cluster separation was weak and reproducibility limited. Organisation-level variance ranged from 4.2% to 12.8%. Except for nurse participation, adjusted sector associations were sensitive to organisational clustering or to individual organisations. Exploratory indirect-effect analyses identified statistical associations involving nurse participation but did not establish mediation. Conclusions: Multidimensional, organisation-sensitive assessments may complement aggregate scores and guide research on structural vulnerabilities and participatory governance.

## 1. Introduction

Nursing practice environments encompass the organisational, managerial, and professional conditions in which nurses provide care. More favourable environments have consistently been associated with better outcomes for nurses, patients, and healthcare organisations, including lower burnout, greater professional retention, and improved quality and safety of care [1,2,3]. The nursing practice environment is therefore increasingly recognised as an organisational determinant of both workforce sustainability and healthcare quality.

The nursing practice environment is a multidimensional construct involving organisational structures, professional relationships, leadership, participation in decision-making, and the availability of resources that support nursing care [4,5,6,7]. This perspective recognises that high-quality nursing care depends not only on individual professional competence but also on organisational conditions that enable nurses to exercise professional judgement, participate in decision-making, collaborate effectively, and provide care with adequate resources. Magnet^®^ hospital principles have similarly emphasised the importance of supportive professional environments for nursing outcomes and organisational performance [8].

Practice environments vary not only across healthcare systems but also between organisations operating within the same system. A comparative study conducted in Portugal and Brazil identified differences in nurses’ perceptions of hospital practice environments and highlighted challenges related to organisational participation, leadership, and resource adequacy [9]. Such variability suggests that broad contextual characteristics may not fully explain nurses’ perceptions and reinforces the importance of considering the healthcare organisation itself as a relevant level of analysis.

The Practice Environment Scale of the Nursing Work Index (PES-NWI) is one of the most widely used instruments for assessing these organisational characteristics [10,11,12,13]. It comprises five dimensions: Nurse Participation in Organisational Affairs; Nursing Foundations for Quality of Care; Nurse Manager Ability, Leadership and Support; Staffing and Resource Adequacy; and Collegial Nurse–Physician Relations. International research has consistently linked these dimensions to relevant professional and organisational outcomes, including nurses’ well-being, perceptions of care quality, and intention to leave [14,15]. However, most applications of the PES-NWI have primarily interpreted dimensions separately or through aggregate classifications of overall practice-environment favourability.

Portuguese evidence has also identified associations between the nursing practice environment, quality of care, and patient safety, particularly in primary healthcare [16], although recent research suggests that contextual adaptations of the PES-NWI may be required to capture organisational specificities across care settings [17]. Despite this growing body of evidence [18], an important question remains insufficiently explored: whether apparently favourable or unfavourable practice environments represent relatively homogeneous organisational experiences or conceal distinct combinations of strengths and vulnerabilities. Favourable clinical and relational foundations may, for example, coexist with limited participation in organisational affairs, insufficient leadership support, or inadequate staffing and resources. Consequently, similar overall PES-NWI scores may reflect substantially different organisational realities. Examining the configuration of dimensions within individual nurses, rather than relying exclusively on aggregate scores, may therefore reveal patterns that conventional overall classifications fail to identify.

A second unresolved issue concerns the level at which differences in nursing practice environments arise. Nurses working within the same healthcare organisation share governance structures, leadership models, staffing policies, resource constraints, and organisational cultures, and their perceptions may therefore be more similar than those of nurses working in different organisations. Consequently, associations attributed to individual professional characteristics or broad organisational categories, such as public versus private sector, may partly reflect organisation-level variation. This distinction is important because sector differences alone offer limited guidance for organisational improvement, whereas identifying modifiable characteristics operating within organisations may provide more actionable evidence. Portuguese studies have predominantly focused on specific organisations or care contexts, limiting opportunities to distinguish individual, professional-, sector-, and organisation-level contributions across multiple healthcare organisations.

Among potentially modifiable organisational characteristics, Nurse Participation in Organisational Affairs may be particularly relevant. Participation reflects opportunities for nurses to contribute to decision-making, governance, policy development, and organisational processes that shape professional practice. Similarly, Staffing and Resource Adequacy represents a structural condition that may enable or constrain the translation of professional standards into everyday care. Examining whether these organisational characteristics statistically account for observed differences in other practice-environment dimensions may help move interpretation beyond descriptive sector comparisons towards potentially actionable organisational mechanisms.

Accordingly, this study aimed to examine the nursing practice environment across Portuguese healthcare organisations from a multidimensional and organisation-sensitive perspective. Specifically, the study sought to: (1) characterise the five PES-NWI dimensions and examine their associations with professional and organisational characteristics; (2) explore within-person configurations of practice-environment dimensions; (3) determine the extent to which perceptions cluster within healthcare organisations and assess whether accounting for this clustering alters estimated sector-related differences; and (4) explore whether Nurse Participation in Organisational Affairs and Staffing and Resource Adequacy statistically account for sector-related differences in selected practice-environment dimensions.

## 2. Methods

### 2.1. Study Design

An observational, multicentre, cross-sectional analytical study was conducted across Portuguese healthcare organisations and reported in accordance with the Strengthening the Reporting of Observational Studies in Epidemiology (STROBE) recommendations. The design enabled the nursing practice environment to be examined at multiple analytical levels, including its dimensional structure, associations with professional and organisational characteristics, within-person configurations of PES-NWI dimensions, and variation attributable to healthcare organisations. Given the cross-sectional design, all identified relationships, including indirect effects, were interpreted as statistical associations rather than causal effects.

### 2.2. Population and Sample

The study was conducted across 29 public and private healthcare organisations located in mainland Portugal and the autonomous regions. Participating organisations predominantly comprised hospital settings, although other care-delivery settings were represented. The target population consisted of registered nurses in active professional practice within participating Portuguese healthcare organisations. A two-stage non-probability convenience sampling strategy was used. First, healthcare organisations that agreed to participate were recruited; second, potentially eligible nurses within those organisations were invited to complete the questionnaire. The final individual-level analytical sample comprised 1004 nurses.

The sample predominantly comprised nurses working in hospital settings. Of the 29 participating organisations, 27 were public and 2 were private (classified according to the sector reported by most of their respondents). The number of participating nurses per organisation ranged from 5 to 169 (median 34; interquartile range 17–48).

Organisational identifiers were obtained from the organisation-specific survey link through which each participant accessed the questionnaire, distributed by each participating organisation, whereas practice sector was recorded through a separate self-reported questionnaire item. Participants were instructed to evaluate the practice environment of the organisation in which they worked at the time of data collection. Consequently, the healthcare-sector coefficients reported in this study reflect individually self-reported practice sector, whereas organisational clustering was modelled using the organisation identifier.

No formal a priori sample-size calculation was performed because all eligible nurses accessible through the participating organisations were invited to participate. The resulting sample of 1004 nurses provided sufficient observations for the planned individual-level multivariable and profile analyses. However, the non-probability sampling strategy limits population-level generalisability, and the number of organisations available for multilevel analyses warrants cautious interpretation of organisation-level estimates.

Registered nurses in active practice at one of the participating organisations and performing direct care, clinical coordination, or nursing management roles were eligible. Nursing students, nurses not in active practice during the data-collection period, and responses not meeting the eligibility criteria or lacking a complete questionnaire were excluded.

### 2.3. Variables

Explanatory variables were organised into sociodemographic, professional, and organisational domains. Sociodemographic variables included age, sex, and geographical region. Professional variables included academic qualifications, postgraduate training, nursing specialty, professional category, role performed, employment status, years of professional practice, years of practice within the current organisation, and area of practice. Organisational variables included healthcare sector (public or private) and, where available, care setting. Distance between residence and workplace was included as an additional professional-characterisation variable.

The primary outcome variables were the mean scores of the five PES-NWI subscales [19]: Nurse Participation in Organisational Affairs (D1), Nursing Foundations for Quality of Care (D2), Nurse Manager Ability, Leadership and Support (D3), Staffing and Resource Adequacy (D4), and Collegial Nurse–Physician Relations (D5). Higher scores indicated more favourable perceptions of the corresponding practice-environment dimension. Overall PES-NWI favourability was used descriptively and to examine whether the multidimensional profiles identified in the profile analysis represented configurations distinct from overall practice-environment level.

Variables entered into the multivariable models were selected on substantive grounds rather than on the basis of bivariate statistical significance. Age and sex were included as basic sociodemographic characteristics; professional category and years of professional practice as indicators of professional position and experience; and healthcare sector and employment status as organisational and contractual characteristics plausibly associated with governance, staffing, and working conditions.

Geographical area (district), although initially considered within the organisational domain, was not included in the final models because it comprised 16 categories, several with very small numbers of participants, and substantially overlapped with the participating organisations. Consequently, geographical effects could not be reliably distinguished from organisation-level variation within the available data. Role performed was also excluded because of its conceptual and empirical overlap with professional category, particularly the nurse-manager category.

Given the strong association between age and years of professional practice, both variables were retained as adjustment covariates, while their individual regression coefficients were not interpreted separately because of substantial multicollinearity. Both variables were retained a priori because they represent conceptually distinct constructs—chronological age reflects life-course and generational characteristics, whereas years of professional practice reflects accumulated professional experience—and because their purpose in the models was confounding adjustment rather than estimation of their own effects. Although substantial multicollinearity may reduce the precision and interpretability of the individual regression coefficients for age and years of professional practice, sensitivity analyses excluding either variable showed minimal changes in the estimated healthcare-sector coefficients. These findings support the stability of the sector-related estimates with respect to the inclusion of these two covariates, while warranting caution in interpreting their individual associations.

Covariates were organised hierarchically into sociodemographic, professional, and organisational blocks to examine their incremental contribution to each PES-NWI dimension. The exact composition of the regression blocks and the corresponding multilevel model specifications are described in Section 2.5.

### 2.4. Data Collection Instrument

The instrument used was the validated Portuguese version of the PES-NWI [19], comprising 31 items distributed across five dimensions: Nurse Participation in Organisational Affairs (9 items), Nursing Foundations for Quality of Care (10 items), Nurse Manager Ability, Leadership and Support (5 items), Staffing and Resource Adequacy (4 items), and Collegial Nurse–Physician Relations (3 items) (Table 1). Items are rated on a four-point Likert scale ranging from 1 (“Strongly disagree”) to 4 (“Strongly agree”), with higher scores reflecting a more favourable perception of the practice environment. The electronic version used fully reproduced the structure, wording, and order of items of the validated Portuguese version. All items were configured as mandatory, so there were no item-level missing data in the submitted questionnaires. Before data collection began, the electronic version of the questionnaire was pre-tested with three nurses external to the study, to assess the clarity of the instructions, comprehension of the items, completion time, and the correct functioning of the electronic platform. No difficulties warranting changes to the instrument were identified.

The Portuguese version of the PES-NWI has previously demonstrated adequate psychometric properties [19,20]. Internal consistency was assessed in the present sample for the overall PES-NWI (Cronbach’s α = 0.85) and each of its five subscales using Cronbach’s alpha. Reliability estimates were interpreted alongside the multidimensional structure of the instrument (Table 2).

The practice environment classification was determined according to the criterion proposed by Lake [21], based on the number of PES-NWI subscales with a mean score above 2.5. According to this criterion, the environment is considered unfavourable when 0 or 1 dimension has a mean above 2.5, mixed when this number is 2 or 3, and favourable when 4 or 5 dimensions exceed this reference value (Table 3). This aggregate classification was used descriptively and as a comparator for the multidimensional profile analysis; it was not treated as a substitute for dimension-specific assessment.

In the present study, Lake’s criterion was applied at two levels: (i) to the sample mean scores of the five subscales, to characterise the overall practice environment of the sample; and (ii) to each participant’s own subscale scores, to classify the environment perceived by each nurse as favourable, mixed, or unfavourable. The individual-level classification was used to describe the distribution of participants and for cross-tabulation with the multidimensional profiles.

The PES-NWI is one of the most widely used instruments internationally for assessing the nursing practice environment, enabling characterisation of the organisational conditions that influence quality of care, professional satisfaction, and outcomes associated with nursing practice [11,22].

### 2.5. Data Collection and Analysis Procedures

Data were collected between 31 January 2023 to 31 March 2023 using an online questionnaire administered through Google Forms. The estimated completion time was 20–30 min. Participating organisations distributed the survey invitation to potentially eligible nurses through institutional email and other internal communication channels. Recruitment and data collection commenced only after approval by the Ethics Committee of the University of Évora and the relevant participating organisations. In each organisation, recruitment was initiated only after the corresponding local approval had been obtained.

Statistical analyses were performed using IBM SPSS Statistics, version 29.0. The analytical approach was structured according to the study objectives and comprised four complementary stages.

Stage 1—Descriptive, comparative, and multivariable analyses.

Participant characteristics and PES-NWI subscale scores were summarised using absolute and relative frequencies for categorical variables and means, standard deviations, minimum values, and maximum values for continuous variables. Differences between two groups were examined using independent-samples t-tests. Homogeneity of variances was assessed using Levene’s test, with Welch’s correction applied when this assumption was violated. Comparisons involving three or more groups were conducted using one-way analysis of variance, followed by Tukey or Games–Howell post hoc tests, as appropriate. Effect sizes were reported using Cohen’s d for two-group comparisons and eta squared (η^2^) for analyses of variance. Associations between continuous variables were examined using Pearson’s correlation coefficient. Distributional assumptions were assessed through inspection of histograms, Q–Q plots, skewness, and kurtosis. When appropriate, equivalent non-parametric analyses were conducted as sensitivity analyses.

Multiple linear regression models were fitted separately for each of the five PES-NWI dimensions to examine the contribution of sociodemographic, professional, and organisational characteristics. Predictors were entered hierarchically according to these domains. Unstandardised coefficients (B), standard errors, standardised coefficients (β), 95% confidence intervals, *p*-values, adjusted R^2^, and changes in R^2^ between successive blocks were reported. Linearity, homoscedasticity, and residual normality were assessed graphically, and multicollinearity was evaluated using tolerance values and variance inflation factors. All tests were two-tailed, with statistical significance set at *p* < 0.05.

Blocks were defined a priori as follows: Block 1, sex and age; Block 2, professional category (reference: generalist nurse) and years of professional practice; Block 3, healthcare sector (private = 1) and employment status (reference: CTFP). The same covariate set was used for all five outcomes and in the corresponding multilevel models. For each block, adjusted R^2^ is reported, whereas ΔR^2^ and the corresponding F-change test refer to changes in unadjusted R^2^. Given the expected overlap between age and years of professional practice, and between professional category and management role, collinearity was examined using tolerance values and variance inflation factors (VIFs).

Analyses addressing objectives 1–3 comprised descriptive and comparative analyses, hierarchical regression models, exploratory profile analysis, and multilevel models. Non-parametric tests were conducted as sensitivity analyses, whereas the indirect-effect analyses addressing objective 4 were considered exploratory. No formal multiplicity adjustment was applied to the primary analyses, which were interpreted with emphasis on effect estimates, their 95% confidence intervals, and the consistency of findings across analytical approaches rather than on statistical significance alone.

To assess the sensitivity of selected findings to multiple testing, a Bonferroni-adjusted significance threshold of α = 0.01 (0.05/5) was applied within specified families of five tests corresponding to the five PES-NWI outcomes. These sensitivity analyses were not intended to control the overall family-wise error rate across all statistical comparisons performed in the study. Findings that did not meet the adjusted threshold were interpreted cautiously, acknowledging the increased risk of false-positive results associated with multiple testing.

Stage 2—Multidimensional profile analysis.

To investigate whether aggregate PES-NWI scores concealed distinct configurations of organisational strengths and vulnerabilities, the five PES-NWI subscale scores were centred within each participant by subtracting the participant-specific mean across all five dimensions from each individual subscale score. These ipsatised scores therefore represented the relative configuration of the practice environment independently of each nurse’s overall level of favourability. K-means clustering was subsequently applied to identify groups of nurses with similar multidimensional profiles. Solutions with two to six clusters were estimated (Euclidean distance; 100 random initial configurations). Candidate solutions were compared using the within-cluster sum of squares (elbow criterion), the average silhouette width, the Calinski–Harabasz index, and the Davies–Bouldin index, together with a minimum cluster size of 10% of the sample, interpretability, and substantive distinctiveness. The stability of the selected solution was assessed by bootstrap resampling (1000 replicates), using the cluster-wise Jaccard similarity coefficient (values ≥ 0.75 indicating stable clusters), and its reproducibility by split-half replication, using the adjusted Rand index. Profile membership was subsequently cross-tabulated with the individual-level PES-NWI favourability classification (Section 2.4) to determine whether the identified configurations provided information beyond overall practice-environment level.

Stage 3—Organisation-level analysis.

To account for the hierarchical structure of the data, with nurses nested within healthcare organisations, linear mixed-effects models with a random intercept for organisation were fitted separately for each of the five PES-NWI dimensions. Intraclass correlation coefficients (ICCs) were estimated from intercept-only (null) models to quantify the proportion of total variance attributable to between-organisation differences.

Adjusted multilevel models included the same covariates as the corresponding ordinary least-squares (OLS) regression models: sex, age, professional category, years of professional practice, healthcare sector, and employment status. This approach enabled comparisons between models with equivalent fixed-effect specifications while accounting for within-organisation dependence in the multilevel analyses.

Multilevel models were estimated using restricted maximum likelihood (REML) in the SPSS MIXED procedure, with denominator degrees of freedom approximated using the Satterthwaite method. Given the relatively small number of organisations (*n* = 29) and unequal cluster sizes, a sensitivity analysis using the Kenward–Roger small-sample correction was conducted in R using the lme4, lmerTest, and pbkrtest packages.

Additionally, a leave-one-organisation-out sensitivity analysis was performed to assess the influence of individual organisations on the estimated healthcare-sector associations. This analysis was particularly relevant because only two participating organisations were predominantly private, limiting the organisational-level information available for public–private comparisons.

Sector-related estimates were therefore interpreted as sample-specific associations rather than generalisable differences between public and private healthcare organisations. A sector association was considered consistent across analytical approaches only when it remained statistically significant in the primary multilevel model, in the Kenward–Roger sensitivity analysis, under the Bonferroni-adjusted threshold, and in all leave-one-organisation-out iterations; otherwise, it was classified as organisation-sensitive.

Stage 4—Exploratory indirect-effect analysis.

Exploratory cross-sectional indirect-effect analyses were conducted to examine the statistical decomposition of associations between healthcare sector and selected PES-NWI dimensions through Nurse Participation in Organisational Affairs or Staffing and Resource Adequacy. These analyses were intended to describe patterns of shared statistical associations rather than to identify causal mechanisms or establish mediation.

Four single-intervening-variable models were specified using Model 4 of the PROCESS macro for IBM SPSS Statistics. Healthcare sector (0 = public; 1 = private) was entered as the explanatory variable; Nurse Participation in Organisational Affairs (D1) or Staffing and Resource Adequacy (D4) as the intervening variable; and Nursing Foundations for Quality of Care (D2) or Collegial Nurse–Physician Relations (D5) as the outcome. No additional covariates were included in these exploratory component models.

For each model, the total association (c) was decomposed into a direct component (c′) and an indirect component (a × b), such that c = c′ + a × b. The indirect component was estimated as the product of the regression coefficients corresponding to the association between healthcare sector and the intervening variable (a) and the conditional association between the intervening variable and the outcome (b). The proportion of the total statistical association represented by the indirect component was calculated as (a × b/c) × 100, where applicable. These proportions were interpreted descriptively and not as percentages of causal effects explained.

Percentile bootstrap 95% confidence intervals for the indirect components were estimated using 3000 individual-level resamples. Because this conventional procedure does not account for the nesting of nurses within healthcare organisations, an additional sensitivity analysis was conducted using a cluster bootstrap with 5000 replicates, in which healthcare organisations were resampled with replacement.

Given the cross-sectional design, the simultaneous measurement of the PES-NWI dimensions, and the absence of additional covariate adjustment, these analyses were considered exploratory statistical decompositions. They do not establish temporal ordering, causal mediation, or the direction of the observed associations.

### 2.6. Ethical Considerations

Before participation, all nurses received information about the study objectives and procedures, the voluntary nature of participation, and the anonymity and confidentiality of the data, and provided electronic informed consent. The study was conducted in accordance with the ethical principles of the Declaration of Helsinki and the Convention on Human Rights and Biomedicine (Oviedo Convention) and was approved by the Ethics Committee of the university to which the principal investigator was affiliated (opinion no. 22110). Personal data were processed in accordance with Regulation (EU) 2016/679 (General Data Protection Regulation—GDPR), with anonymisation procedures and access restricted to the research team.

## 3. Results

### 3.1. Sample Characteristics

The analytical sample comprised 1004 nurses from 29 Portuguese healthcare organisations (27 public and 2 private). Most participants were female (81.9%) and worked in the public sector (90.8%). Sociodemographic and professional characteristics are presented in Table 4, and descriptive statistics for continuous variables are reported in Table 5.

The 29 participating organisations comprised 27 predominantly public and 2 predominantly private organisations. The number of respondents per organisation ranged from 5 to 169 (median 34; interquartile range 17–48), and 4 organisations contributed fewer than 10 respondents. Private-sector participants (n = 92) were distributed across 8 organisations (82 in the two predominantly private organisations and 10 in six predominantly public organisations).

The mean age was 42.58 ± 9.76 years, with a mean length of professional practice of 19.44 ± 9.97 years and a mean length of time at the current organisation of 16.53 ± 10.51 years (Table 5).

### 3.2. Descriptive, Comparative, and Multivariable Analyses

Mean scores for the five PES-NWI dimensions are presented in Table 6.

At the sample level, two of the five dimensions (D2 and D5) had mean values above 2.5; according to Lake’s criterion, the overall practice environment of the sample was therefore classified as mixed. When the same criterion was applied to each participant’s own subscale scores, 293 nurses (29.2%) perceived a favourable environment, 389 (38.7%) a mixed environment, and 322 (32.1%) an unfavourable environment. The sample-level classification therefore summarises the central tendency of the sample and does not imply that all nurses perceived a mixed environment.

Regarding the normality assumptions, the Kolmogorov–Smirnov and Shapiro–Wilk tests were statistically significant for all dimensions (*p* < 0.001 in all cases), indicating departure from normality. Skewness and kurtosis values fell within acceptable limits (|values| < 1) for all dimensions, with the exception of D5, which showed slightly more pronounced values, although still within the |1| limit. Non-parametric sensitivity analysis, using the Mann–Whitney U test (for sector) and the Kruskal–Wallis test (for professional category), confirmed the same statistical significance and the same direction of results obtained with the parametric tests across all dimensions (Table 7 and Table 8).

Comparisons between public- and private-sector nurses are presented in Table 9, and differences according to professional category in Table 10.

Correlations between PES-NWI dimensions are presented in Table 11a.

The full hierarchical regression results, including, for each block, R^2^, adjusted R^2^, ΔR^2^ and the corresponding F test for change, together with the final model coefficients (B, SE, β, 95% CI and *p*), are presented below in Table 11b–g.

Among the five PES-NWI dimensions, Nursing Foundations for Quality of Care showed the highest mean score (M = 2.78, SD = 0.51), followed by Collegial Nurse–Physician Relations (M = 2.70, SD = 0.61), both exceeding the reference value of 2.5. Nurse Participation in Organisational Affairs (M = 2.27, SD = 0.56) and Staffing and Resource Adequacy (M = 2.28, SD = 0.61) showed the lowest mean scores. Given the large sample size and graphical inspection of the distributions, parametric analyses were retained. In unadjusted comparisons, nurses working in private-sector organisations reported higher mean scores across all five PES-NWI dimensions than nurses working in public-sector organisations (Table 9). Differences were statistically significant for Nurse Participation in Organisational Affairs, Nursing Foundations for Quality of Care, Nurse Manager Ability, Leadership and Support, and Collegial Nurse–Physician Relations (all *p* < 0.001), as well as for Staffing and Resource Adequacy (*p* = 0.003). Effect sizes were small to moderate in magnitude, ranging from |d| = 0.33 to |d| = 0.55, with higher mean scores observed in the private-sector group. These comparisons did not account for the clustering of nurses within healthcare organisations.

**Figure 1:**
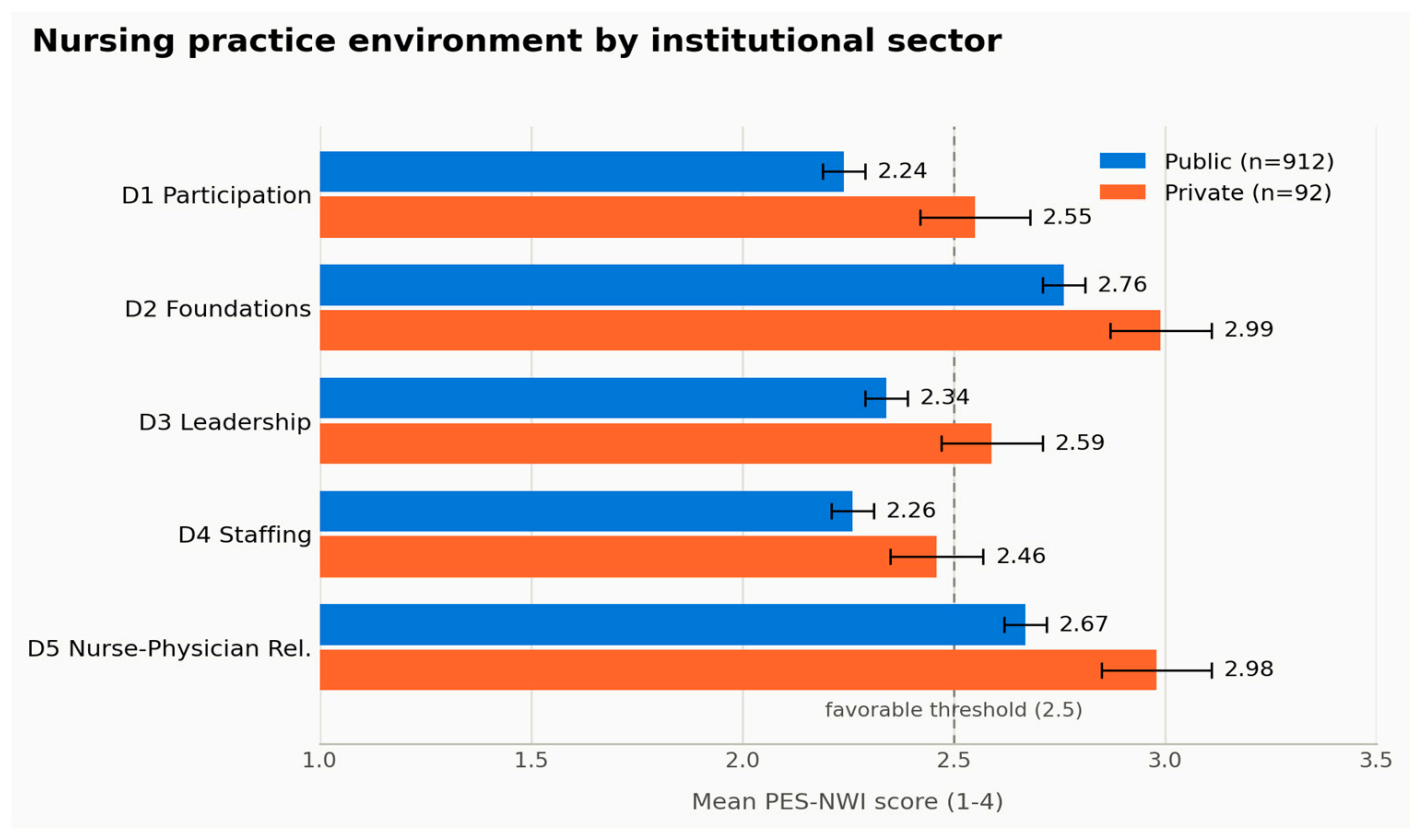
Mean PES-NWI subscale scores by practice sector, with 95% confidence intervals.

Sector-specific estimates were markedly less precise for the private sector (n = 92) than for the public sector (n = 912). For example, the 95% confidence interval for Nurse Participation in Organisational Affairs was 2.20–2.28 in the public sector and 2.43–2.67 in the private sector. Mean differences (private − public) and their 95% confidence intervals were 0.31 [0.19, 0.43] for D1, 0.23 [0.12, 0.34] for D2, 0.25 [0.11, 0.39] for D3, 0.20 [0.07, 0.33] for D4, and 0.31 [0.18, 0.44] for D5. All unadjusted sector differences remained statistically significant under the Bonferroni-adjusted threshold (α = 0.01) (Figure 1).

**Figure 2:**
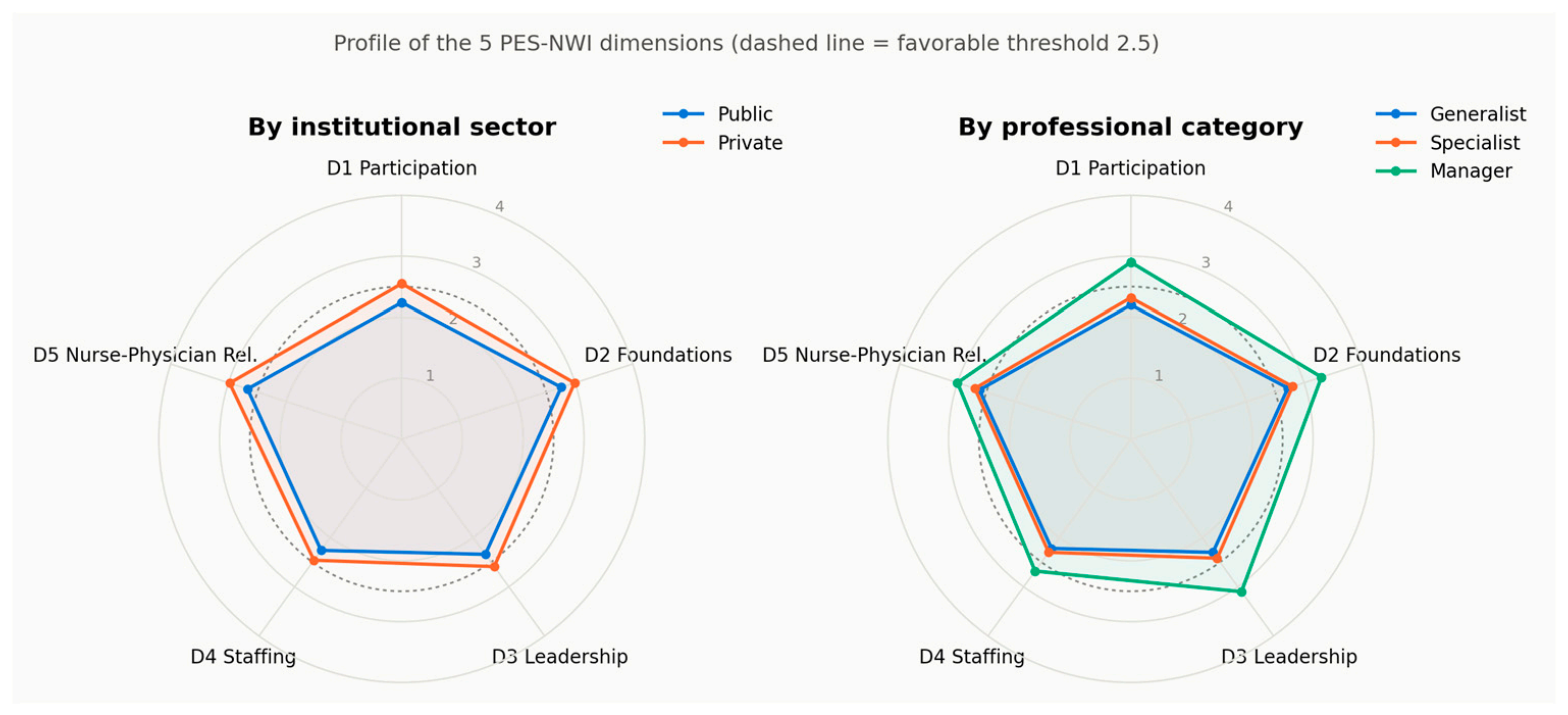
Radar profile of the five PES-NWI dimensions by practice sector and by professional category. All five PES-NWI dimensions were positively correlated (all *p* < 0.001), with coefficients ranging from r = 0.361 for Staffing and Resource Adequacy with Collegial Nurse–Physician Relations to r = 0.755 for Nurse Participation in Organisational Affairs with Nurse Manager Ability, Leadership and Support (Table 11a; Figure 3).

Significant differences across professional categories were observed for all five PES-NWI dimensions (Table 10), although effect sizes were small (η^2^ = 0.011–0.053). Post hoc comparisons (Games–Howell) indicated that nurse managers reported higher scores than generalist nurses for all five dimensions (adjusted *p* < 0.001 for D1–D4; adjusted *p* = 0.004 for D5) and higher scores than specialist nurses for all five dimensions (adjusted *p* < 0.001 for D1–D3; *p* = 0.006 for D4; *p* = 0.030 for D5). In contrast, the differences between specialist and generalist nurses were small (0.03–0.09 points) and were not statistically significant for any dimension (adjusted *p* = 0.072–0.779); they should therefore be interpreted descriptively. These unadjusted post hoc comparisons are distinct from the covariate-adjusted regression estimates reported below (Table 11c–g), in which the specialist–generalist contrast reached nominal statistical significance only for D1 (*p* = 0.042) and D4 (*p* = 0.043), and neither met the Bonferroni-adjusted threshold (α = 0.01) (Figure 2).

**Table 11:** (**a**) Pearson correlation matrix between PES-NWI subscales. (**b**) Model summary by block. (**c**) Final model (Block 3)—D1, Nurse Participation in Organisational Affairs. (**d**) Final model (Block 3)—D2, Nursing Foundations for Quality of Care. (**e**) Final model (Block 3)—D3, Nurse Manager Ability, Leadership and Support. (**f**) Final model (Block 3)—D4, Staffing and Resource Adequacy. (**g**) Final model (Block 3)—D5, Collegial Nurse–Physician Relations.

(**a**)							
			**D1**	**D2**	**D3**	**D4**	**D5**
D1 Nurse Participation in Organisational Affairs			—				
D2 Nursing Foundations for Quality of Care			0.692 **	—			
D3 Nurse Manager Ability, Leadership and Support			0.755 **	0.636 **	—		
D4 Staffing and Resource Adequacy			0.538 **	0.453 **	0.527 **	—	
D5 Collegial Nurse–Physician Relations			0.472 **	0.476 **	0.433 **	0.361 **	—
(**b**)							
**Dimension**	**Block 1 R^2^ (adj.)**	**Block 2 R^2^ (adj.)**	**ΔR^2^ (B1 → B2)**	**Block 3 R^2^ (adj.)**	**ΔR^2^ (B2 → B3)**	**F Change (B2 → B3)**	***p* (Change)**
D1 Nurse Participation in Organisational Affairs	0.015	0.052	0.039	0.080	0.032	F(4, 994) = 8.73	<0.001
D2 Nursing Foundations for Quality of Care	0.017	0.051	0.036	0.069	0.021	F(4, 994) = 5.76	<0.001
D3 Nurse Manager Ability, Leadership and Support	0.010	0.045	0.038	0.059	0.018	F(4, 994) = 4.72	<0.001
D4 Staffing and Resource Adequacy	0.013	0.021	0.010	0.038	0.021	F(4, 994) = 5.39	<0.001
D5 Collegial Nurse–Physician Relations	0.008	0.010	0.005	0.036	0.030	F(4, 994) = 7.76	<0.001
(**c**)							
**Variable**			**B**	**SE**	**β**	**95% CI**	***p***
Professional category: Manager (vs. Generalist)			0.508	0.082	0.212	[0.348, 0.668]	<0.001
Professional category: Specialist (vs. Generalist)			0.078	0.038	0.066	[0.003, 0.154]	0.042
Female sex (vs. male)			0.074	0.044	0.051	[−0.013, 0.161]	0.095
Age			0.005	0.006	0.093	[−0.007, 0.018]	0.405
Years of professional practice			−0.001	0.006	−0.020	[−0.014, 0.011]	0.858
Private sector (vs. public)			0.325	0.061	0.167	[0.205, 0.445]	<0.001
Employment status: CIT-TC (vs. CTFP)			0.167	0.122	0.046	[−0.071, 0.406]	0.169
Employment status: CIT-TI (vs. CTFP)			0.007	0.048	0.006	[−0.088, 0.101]	0.891
Employment status: Other (vs. CTFP)			0.098	0.100	0.032	[−0.099, 0.296]	0.327
(**d**)							
**Variable**			**B**	**SE**	**β**	**95% CI**	***p***
Professional category: Manager (vs. Generalist)			0.449	0.075	0.204	[0.301, 0.597]	<0.001
Professional category: Specialist (vs. Generalist)			0.055	0.035	0.050	[−0.015, 0.124]	0.124
Female sex (vs. male)			0.140	0.041	0.105	[0.060, 0.220]	0.001
Age			0.000	0.006	−0.008	[−0.012, 0.011]	0.945
Years of professional practice			0.003	0.006	0.053	[−0.009, 0.014]	0.641
Private sector (vs. public)			0.249	0.056	0.140	[0.138, 0.360]	<0.001
Employment status: CIT-TC (vs. CTFP)			0.095	0.112	0.028	[−0.125, 0.315]	0.396
Employment status: CIT-TI (vs. CTFP)			−0.006	0.045	−0.005	[−0.093, 0.082]	0.899
Employment status: Other (vs. CTFP)			0.063	0.093	0.022	[−0.119, 0.245]	0.497
(**e**)							
**Variable**			**B**	**SE**	**β**	**95% CI**	***p***
Professional category: Manager (vs. Generalist)			0.606	0.098	0.214	[0.414, 0.797]	<0.001
Professional category: Specialist (vs. Generalist)			0.057	0.046	0.041	[−0.033, 0.147]	0.215
Female sex (vs. male)			−0.008	0.053	−0.005	[−0.111, 0.096]	0.880
Age			0.009	0.008	0.126	[−0.006, 0.024]	0.264
Years of professional practice			−0.009	0.008	−0.136	[−0.024, 0.006]	0.238
Private sector (vs. public)			0.261	0.073	0.114	[0.118, 0.404]	<0.001
Employment status: CIT-TC (vs. CTFP)			0.068	0.145	0.016	[−0.217, 0.353]	0.641
Employment status: CIT-TI (vs. CTFP)			−0.100	0.058	−0.075	[−0.213, 0.013]	0.084
Employment status: Other (vs. CTFP)			0.051	0.120	0.014	[−0.184, 0.287]	0.669
(**f**)							
**Variable**			**B**	**SE**	**β**	**95% CI**	***p***
Professional category: Manager (vs. Generalist)			0.238	0.091	0.091	[0.059, 0.417]	0.009
Professional category: Specialist (vs. Generalist)			0.087	0.043	0.067	[0.003, 0.171]	0.043
Female sex (vs. male)			−0.004	0.049	−0.003	[−0.101, 0.092]	0.934
Age			−0.004	0.007	−0.058	[−0.018, 0.010]	0.611
Years of professional practice			0.009	0.007	0.154	[−0.005, 0.023]	0.184
Private sector (vs. public)			0.227	0.068	0.107	[0.093, 0.360]	0.001
Employment status: CIT-TC (vs. CTFP)			0.136	0.135	0.034	[−0.130, 0.401]	0.317
(**g**)							
**Variable**			**B**	**SE**	**β**	**95% CI**	***p***
Professional category: Manager (vs. Generalist)			0.174	0.091	0.067	[−0.004, 0.353]	0.056
Professional category: Specialist (vs. Generalist)			0.061	0.043	0.048	[−0.023, 0.145]	0.152
Female sex (vs. male)			0.069	0.049	0.043	[−0.028, 0.165]	0.162
Age			0.006	0.007	0.100	[−0.008, 0.020]	0.384
Years of professional practice			0.002	0.007	0.026	[−0.012, 0.016]	0.825
Private sector (vs. public)			0.320	0.068	0.152	[0.187, 0.454]	<0.001
Employment status: CIT-TC (vs. CTFP)			0.062	0.135	0.016	[−0.204, 0.328]	0.646
Employment status: CIT-TI (vs. CTFP)			0.079	0.054	0.064	[−0.026, 0.185]	0.140
Employment status: Other (vs. CTFP)			0.210	0.112	0.062	[−0.010, 0.430]	0.061

(**a**) Note. ** *p* < 0.001. (**b**) Note. D1 = Nurse Participation in Organisational Affairs; D2 = Nursing Foundations for Quality of Care; D3 = Nurse Manager Ability, Leadership and Support; D4 = Staffing and Resource Adequacy; D5 = Collegial Nurse–Physician Relations. Block 1 included sex and age (2 predictors); Block 2 additionally included professional category and years of professional practice (5 parameters in total); and Block 3 additionally included healthcare sector and employment status (9 parameters in total). The R^2^ values reported for each block are adjusted R^2^ values, whereas ΔR^2^ represents the change in unadjusted R^2^. F-change statistics refer to the comparison between Blocks 2 and 3, with 4 and 994 degrees of freedom, respectively. All models included 1004 participants. (**g**) CTFP = open-ended public-sector employment contract; CIT-TI = open-ended individual employment contract; CIT-TC = fixed-term individual employment contract. Note. All final models included sex, age, professional category, years of professional practice, healthcare sector, and employment status (n = 1004); coefficients, standard errors, 95% confidence intervals, standardised coefficients, and *p*-values in each table derive from the same fitted model. Coefficients for age and years of professional practice are small and are reported rounded to three decimals; minor apparent discrepancies between B/SE and the corresponding *p*-values for these variables reflect rounding. Confidence intervals were computed as B ± t(0.975; 994) × SE.

Hierarchical linear regression models were fitted separately for each PES-NWI dimension using three blocks: Block 1, sex and age; Block 2, professional category (reference: generalist nurse) and years of professional practice; and Block 3, healthcare sector (private = 1) and employment status (reference: CTFP). Geographical area was not included in the final models, as explained in Section 2.3. Model summaries are presented in Table 11b.

Collinearity diagnostics revealed substantial multicollinearity between age and years of professional practice (r = 0.96; VIF = 13.6 and 14.1, respectively; tolerance ≈ 0.07), whereas all other predictors had VIF ≤ 1.98 (tolerance ≥ 0.50). Both variables were retained as adjustment covariates, but their individual regression coefficients were not interpreted independently because of their substantial overlap. Sensitivity analyses excluding either age or years of professional practice showed that the estimated healthcare-sector coefficients changed by no more than 0.003, suggesting that the sector-related estimates were relatively insensitive to the inclusion of both variables. However, this finding does not exclude potential instability in the estimated individual effects of age and professional experience.

The addition of Block 3 was associated with a statistically significant increase in explained variance across all five PES-NWI dimensions (ΔR^2^ = 0.018–0.032, all *p* ≤ 0.001). Nevertheless, the final models explained only 3.6–8.0% of the variance (adjusted R^2^), indicating that most of the variability in nurses’ perceptions remained unexplained by the included covariates.

In the final models (Table 11c–g), which did not account for the clustering of nurses within organisations (see Section 3.4), private-sector employment was positively associated with all five PES-NWI dimensions (β = 0.107–0.167, all *p* ≤ 0.001). Compared with generalist nurses, nurse managers reported higher scores for D1–D4 (β = 0.091–0.213, *p* ≤ 0.009), whereas the association for D5 did not reach statistical significance (β = 0.067, *p* = 0.056). Sex, age, years of professional practice, and employment status did not show consistent associations across dimensions. However, the individual coefficients for age and years of professional practice should be interpreted cautiously because of substantial multicollinearity.

When a Bonferroni-adjusted significance threshold of α = 0.01 was applied within the specified families of tests, the sector associations and manager–generalist differences for D1–D4 remained statistically significant. In contrast, specialist–generalist differences for D1 (*p* = 0.042) and D4 (*p* = 0.043), and the association between the “other” employment-status category and D4 (*p* = 0.029), did not meet the adjusted threshold.

### 3.3. Multidimensional Profile Analysis

Candidate solutions comprising two to six clusters were evaluated using complementary indices of cluster separation and compactness, minimum cluster size, and substantive interpretability (Table 12a). No single solution consistently outperformed the alternatives across all quantitative criteria. The two-cluster solution yielded the highest average silhouette width (0.219) and Calinski–Harabasz index (316.1), whereas the five-cluster solution had the lowest Davies–Bouldin index (1.342). The three-cluster solution was retained as an exploratory compromise between statistical performance, parsimony, cluster size, and interpretability. It showed a reduction in within-cluster sum of squares consistent with an elbow around three clusters, a lower Davies–Bouldin index than the two- and four-cluster solutions, and relatively balanced cluster sizes, with each profile comprising more than 25% of the sample.

Internal stability and reproducibility analyses indicated important limitations. Bootstrap resampling (1000 replicates) yielded cluster-wise Jaccard coefficients of 0.72, 0.67, and 0.64 for Profiles 1, 2, and 3, respectively, suggesting moderate to limited stability. Split-half replication across 100 random splits yielded a median adjusted Rand index of 0.31 (range: 0.25–0.82), suggesting limited reproducibility of the cluster solution. The estimated prevalence of Profile 1 was 36.6%, with a bootstrap 95% percentile interval of 25.5–40.2%. Together with the low average silhouette width (0.212), these findings indicate that the profiles are not sharply separated and should be interpreted as exploratory descriptive configurations rather than stable or externally validated categories. In practical terms, this instability implies that the assignment of individual nurses to a given profile is uncertain, that the estimated profile prevalences including the 36.6% for Profile 1 are imprecise, and that the boundaries between profiles are not sharply defined. For this reason, profile membership was not used as an explanatory or outcome variable in subsequent analyses, and the profiles are not presented as robust or clearly distinct groups.

**Figure 4:**
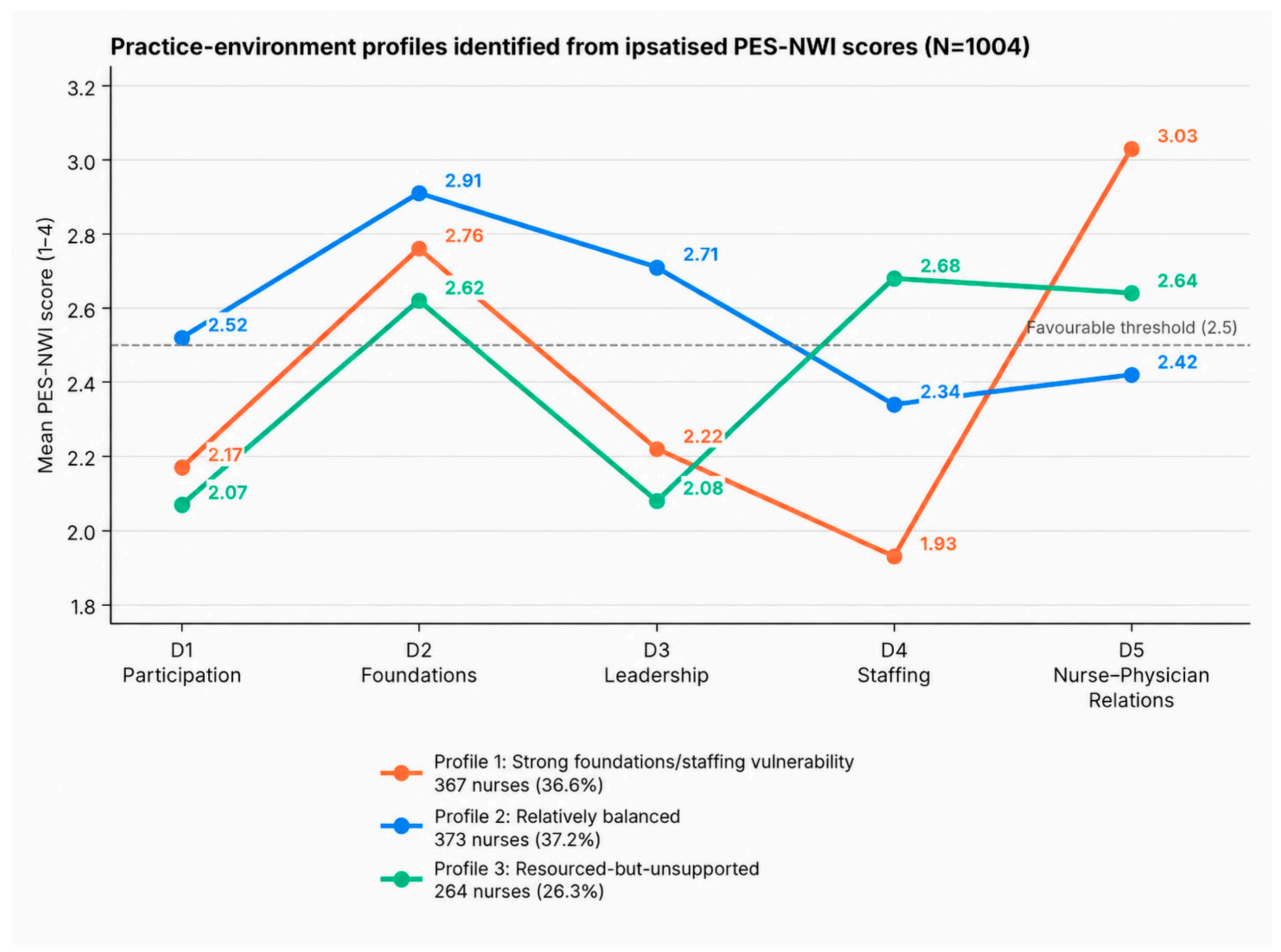
Absolute mean scores across the five PES-NWI dimensions for the three exploratory profiles identified using ipsatised scores. Note. The figure presents absolute mean subscale scores (1–4), whereas profile membership was determined using participant-centred (ipsatised) scores. The dashed horizontal line indicates the PES-NWI reference value of 2.5 and does not, by itself, determine overall practice-environment favourability. Profile labels describe relative patterns of strengths and vulnerabilities and do not represent externally validated categories. Profile 1: strong foundations/staffing vulnerability (n = 367); Profile 2: relatively balanced (n = 373); Profile 3: resourced-but-unsupported (n = 264).

The exploratory cluster analysis based on ipsatised PES-NWI scores yielded a three-profile solution reflecting different patterns of relative strengths and vulnerabilities across the five dimensions (Table 12b; Figure 4). Given the limited cluster separation and moderate internal stability, these profiles should be interpreted as descriptive patterns within the present sample rather than as clearly differentiated or externally validated categories of nursing practice environments.

Profile 1 (strong foundations/staffing vulnerability profile) comprised 367 nurses (36.6%) and was characterised by relatively stronger Collegial Nurse–Physician Relations and Nursing Foundations for Quality of Care, alongside a pronounced relative weakness in Staffing and Resource Adequacy. This profile showed the largest within-profile range (1.10), indicating greater variation between its highest- and lowest-scoring dimensions.

Profile 2 (relatively balanced profile) comprised 373 nurses (37.2%) and exhibited a less pronounced contrast between dimensions (within-profile range = 0.57). Nursing Foundations for Quality of Care was the highest-scoring dimension, whereas Staffing and Resource Adequacy remained relatively lower. Although this profile had the highest mean score across the five dimensions (2.58), its designation reflects the relative configuration of dimension scores rather than an assumption of uniformly favourable practice environments.

Profile 3 (resourced-but-unsupported profile) comprised 264 nurses (26.3%) and showed a contrasting configuration, characterised by relatively higher Staffing and Resource Adequacy and lower Nurse Participation in Organisational Affairs and Nurse Manager Ability, Leadership and Support.

Cross-tabulation with the individual-level PES-NWI favourability classification (Table 12c) showed that Profile 1 included nurses reporting unfavourable (n = 107; 29.2%), mixed (n = 192; 52.3%), and favourable (n = 68; 18.5%) practice environments. This indicates that the relative configuration identified in Profile 1 was not restricted to nurses reporting an overall unfavourable practice environment, highlighting the distinction between relative dimensional patterns and absolute levels of perceived favourability.

### 3.4. Organisation-Level Analysis

Random-intercept multilevel models revealed modest but relevant variation in PES-NWI scores between healthcare organisations (Table 13). Intraclass correlation coefficients (ICCs), estimated from intercept-only models, ranged from 4.2% for Staffing and Resource Adequacy to 12.8% for Nursing Foundations for Quality of Care. These findings indicate that the proportion of total variance represented by the between-organisation variance component differed across practice-environment dimensions. The ICCs reflect the statistical clustering of nurses’ perceptions within organisations and should not be interpreted as evidence of causal organisational effects.

Compared with equivalent ordinary least-squares (OLS) regression models, the adjusted multilevel models yielded larger standard errors for the healthcare-sector coefficients across all five PES-NWI dimensions, with multilevel-to-OLS standard-error ratios ranging from 1.23 to 1.81. The largest difference was observed for Nursing Foundations for Quality of Care (D2), for which the sector coefficient decreased from B = 0.249 (SE = 0.056, *p* < 0.001) in the OLS model to B = 0.170 (SE = 0.102, 95% CI [−0.033, 0.374], *p* = 0.100) in the multilevel model. Thus, the association between healthcare sector and this dimension was no longer statistically significant at the conventional 0.05 threshold after accounting for the hierarchical structure of the data. These differences illustrate the importance of considering organisational clustering when estimating sector-related associations, although they do not establish that healthcare sector independently determines nurses’ perceptions of the practice environment.

In the sensitivity analysis using the Kenward–Roger small-sample correction, the *p*-values for the healthcare-sector association were <0.001 (D1), 0.107 (D2), 0.048 (D3), 0.030 (D4), and 0.003 (D5). The pattern of statistical significance at the conventional 0.05 threshold was unchanged compared with the primary multilevel models. However, the associations for Nurse Manager Ability, Leadership and Support (D3) and Staffing and Resource Adequacy (D4) remained close to this threshold and should be interpreted cautiously.

The leave-one-organisation-out sensitivity analysis provided further evidence of uncertainty in the sector-related estimates. Although the estimated sector coefficients remained positive across all iterations, their statistical significance varied according to the organisation excluded. The association for Nurse Participation in Organisational Affairs (D1) remained statistically significant in all 29 iterations, whereas that for Nursing Foundations for Quality of Care (D2) was non-significant in 26 iterations. The association for Nurse Manager Ability, Leadership and Support (D3) was non-significant in 14 iterations. For Staffing and Resource Adequacy (D4) and Collegial Nurse–Physician Relations (D5), the exclusion of one predominantly private organisation resulted in non-significant associations (*p* = 0.175 and *p* = 0.121, respectively).

When a Bonferroni-adjusted significance threshold of 0.01 was applied across the five dimensions, only the sector associations for Nurse Participation in Organisational Affairs (D1) and Collegial Nurse–Physician Relations (D5) remained statistically significant in the primary multilevel models.

Taken together, these sensitivity analyses indicate that sector-related associations, particularly those involving leadership, staffing, and collegial relations, were sensitive to analytical assumptions and the inclusion of individual organisations. Given the limited number of predominantly private organisations, these findings should be interpreted as sample-specific associations rather than robust or generalisable differences between healthcare sectors.

Table 14b summarises the healthcare-sector associations across analytical approaches, distinguishing findings that were consistent across analyses from organisation-sensitive results. Only the association between healthcare sector and Nurse Participation in Organisational Affairs (D1) met all the predefined consistency criteria. The associations for D2–D5 were organisation-sensitive: the D2 association was no longer statistically significant after accounting for organisational clustering, and the D3, D4, and D5 associations either did not meet the Bonferroni-adjusted threshold or lost statistical significance when individual organisations were excluded.

### 3.5. Exploratory Indirect-Effect Analysis

Exploratory indirect-effect analyses were conducted to examine statistical associations between healthcare sector and selected PES-NWI dimensions through Nurse Participation in Organisational Affairs (D1) and Staffing and Resource Adequacy (D4) (Table 15).

The largest estimated indirect component was observed for the association between healthcare sector and Nursing Foundations for Quality of Care (D2) through Nurse Participation in Organisational Affairs (D1), with an unstandardised estimate of 0.194 (95% percentile bootstrap CI [0.113, 0.275]). The corresponding direct association was 0.037 (*p* = 0.370), and the estimated total association was 0.231.

An indirect component through Nurse Participation in Organisational Affairs (D1) was also observed for Collegial Nurse–Physician Relations (D5), with an estimate of 0.154 (95% CI [0.089, 0.223]). Smaller indirect components through Staffing and Resource Adequacy (D4) were estimated for Nursing Foundations for Quality of Care (D2) (0.075, 95% CI [0.025, 0.127]) and Collegial Nurse–Physician Relations (D5) (0.071, 95% CI [0.024, 0.121]).

The proportions of the total statistical associations represented by the indirect components ranged from 23.3% (Sector → D4 → D5) to 84.0% (Sector → D1 → D2) across the four models (Table 15). These proportions describe model-specific statistical decompositions and should not be interpreted as the proportion of a causal effect explained by the intervening variables.

In the cluster-bootstrap sensitivity analysis, which resampled healthcare organisations rather than individual nurses, the estimated indirect component through Nurse Participation in Organisational Affairs (D1) for Nursing Foundations for Quality of Care (D2) was 0.194 (95% CI [0.059, 0.273]). The corresponding confidence intervals for the remaining pathways were [0.049, 0.213] for D1–D5, [0.020, 0.180] for D4–D2, and [0.018, 0.173] for D4–D5. All four cluster-bootstrap confidence intervals excluded zero, although this does not establish causal mediation or eliminate uncertainty arising from the limited number and composition of participating organisations.

Given the cross-sectional design, the simultaneous measurement of the PES-NWI dimensions, and the absence of covariate adjustment in these models, the findings should be interpreted exclusively as exploratory statistical associations. They do not establish temporal sequences, causal pathways, or mediation mechanisms.

## 4. Discussion

Three main findings emerged from this multicentre study. First, the nursing practice environment was characterised by an uneven multidimensional configuration, and exploratory person-centred profile analysis suggested that this pattern may not be fully captured by aggregate favourability alone: more than one-third of nurses were assigned to an exploratory profile (strong foundations/staffing vulnerability profile; Profile 1), characterised by relatively favourable care-quality foundations and nurse–physician relations alongside markedly lower staffing and resource adequacy scores. Second, nurses’ perceptions showed meaningful clustering within healthcare organisations, and accounting for this hierarchical structure altered the estimated association between healthcare sector and some practice-environment dimensions. Third, in exploratory cross-sectional analyses, Nurse Participation in Organisational Affairs was statistically associated with a substantial share of the sector-related difference in Nursing Foundations for Quality of Care, although these analyses do not establish mediation. Together, these findings suggest that nursing practice environments are better understood as multidimensional and organisation-sensitive phenomena than through aggregate scores or broad public–private comparisons alone. The coexistence of favourable perceptions of care-quality foundations and nurse–physician relations with less favourable perceptions of participation, leadership support, and staffing adequacy constitutes a central feature of the findings. We interpret this as an organisational paradox: professional and relational conditions perceived as supportive of quality care may coexist with structural conditions perceived as substantially less favourable. Previous research has established that the nursing practice environment comprises interrelated but distinct professional, relational, leadership, participatory, and resource dimensions [22,23,24,25,26,27,28]. The present findings extend this perspective by showing that these dimensions may form markedly uneven configurations within individual nurses, rather than simply varying together along a single continuum from unfavourable to favourable.

Nursing Foundations for Quality of Care was the most favourably rated dimension, suggesting that nurses generally perceived the presence of professional standards and organisational foundations supportive of quality-oriented nursing practice. Similar findings have been reported in previous studies [28]. Importantly, however, the PES-NWI captures perceived practice conditions rather than actual clinical performance; these scores should therefore not be interpreted as direct evidence of care quality, patient safety, or patient outcomes. Collegial Nurse–Physician Relations was also rated relatively favourably, indicating that positive interprofessional relationships may remain an organisational resource even when other structural aspects of the practice environment are perceived less favourably. This distinction is relevant because it reinforces the multidimensional nature of the practice environment: favourable relational conditions do not necessarily imply similarly favourable staffing, participation, or leadership conditions [26].

Staffing and Resource Adequacy was among the least favourably rated dimensions, consistent with evidence linking resource constraints and less favourable practice environments to workload, missed nursing care, burnout, and intention to leave [29,30,31]. Its relevance was further reinforced by the profile analysis: staffing adequacy was the lowest-scoring dimension in both the strong foundations/staffing vulnerability profile (Profile 1) and the relatively balanced profile (Profile 2). This finding suggests that staffing vulnerability may remain present even among nurses whose overall assessment of other practice-environment dimensions is comparatively positive. However, because objective nurse-to-patient ratios, workload, patient dependency, skill mix, and staffing-distribution indicators were not collected, the present study cannot determine which specific workforce mechanisms underlie these perceptions.

Nurse Participation in Organisational Affairs was also rated relatively unfavourably. This dimension captures nurses’ opportunities to contribute to organisational decision-making, policy development, governance, and processes affecting professional practice. Previous studies have associated greater participation with empowerment, professional autonomy, and engagement [32,33]. In the present study, exploratory indirect-effect analyses identified a positive indirect statistical component involving participation in the association between healthcare sector and Nursing Foundations for Quality of Care. This finding suggests that perceived participation and perceived foundations for quality care are interrelated within the observed sector-associated patterns. However, the cross-sectional design, simultaneous measurement of PES-NWI dimensions, and absence of covariate adjustment prevent conclusions regarding temporal ordering, causal mediation, or the independent explanatory contribution of participation. Furthermore, these associations may reflect shared organisational characteristics or common sources of variation in nurses’ perceptions. Accordingly, participation should be regarded as a potentially relevant organisational characteristic for further investigation rather than an established mechanism underlying sector differences. Future longitudinal and intervention studies should examine whether changes in participatory governance, representation of direct-care nurses in decision-making, and professional involvement are associated with subsequent improvements in nursing practice environments.

Nurse Manager Ability, Leadership and Support also remained below the favourable reference value, reinforcing the broader pattern in which structural and organisational-support dimensions were less favourably perceived than clinical and relational dimensions. Previous research has highlighted the importance of supportive and empowering leadership for professional autonomy, job satisfaction, and healthy practice environments [34,35,36]. Although the present study did not directly assess specific leadership behaviours, the findings support the inclusion of leadership, communication, professional recognition, feedback, and support for professional development in future organisational assessments.

Unadjusted analyses showed higher PES-NWI scores among private-sector nurses across all five dimensions, with small-to-moderate effect sizes. However, the multilevel findings suggest that these differences require cautious interpretation. Intraclass correlation coefficients indicated that between-organisation variation represented 4.2–12.8% of the total variance across PES-NWI dimensions. Compared with the corresponding ordinary least-squares models, the adjusted multilevel models produced larger standard errors for the healthcare-sector coefficients. For Nursing Foundations for Quality of Care, the sector association was no longer statistically significant at the conventional 0.05 threshold after accounting for organisational clustering. These findings highlight the importance of considering the hierarchical structure of the data when examining sector-related associations.

The interpretation of sector differences is further constrained by the composition of the sample. Only 92 nurses (9.2%) worked in the private sector, and 82 of these participants were concentrated in two predominantly private organisations. Consequently, the estimated sector associations may partly reflect characteristics of the specific organisations included in the study rather than differences consistently attributable to healthcare sector. Potentially relevant characteristics include governance arrangements, staffing policies, leadership practices, workforce composition, and organisational culture, none of which were sufficiently characterised to determine their independent contributions. Previous public–private comparisons have also highlighted the context-dependent nature of nursing work environments and professional satisfaction [37,38], while the specific organisational and governance characteristics of the Portuguese healthcare system warrant consideration [39].

The sensitivity analyses reinforced the need for caution. Although the Kenward–Roger correction did not change the pattern of statistical significance at the 0.05 threshold, the associations for Nurse Manager Ability, Leadership and Support and Staffing and Resource Adequacy remained close to that threshold. Furthermore, the leave-one-organisation-out analysis showed that, although sector coefficients remained positive, statistical significance was sensitive to the exclusion of individual organisations. In particular, the associations for Staffing and Resource Adequacy and Collegial Nurse–Physician Relations were no longer statistically significant following the exclusion of one predominantly private organisation, while the association for Nurse Manager Ability, Leadership and Support was non-significant in approximately half of the iterations. These findings indicate that some sector-related associations were dependent on the organisational composition of the sample. Of the five sector associations, only that involving Nurse Participation in Organisational Affairs was consistent across all analytical approaches; the remaining associations should be regarded as organisation-sensitive (Table 14a). Therefore, the present results should not be interpreted as evidence that private healthcare organisations generally provide more favourable nursing practice environments than public organisations in Portugal. Rather, they support the importance of examining organisational heterogeneity and contextual characteristics in future studies. Professional category was also associated with perceptions of the practice environment, with nurse managers reporting more favourable scores than both generalist and specialist nurses, whereas specialist and generalist nurses reported similar scores. However, effect sizes were small, indicating limited practical differentiation between professional categories. Differences may reflect variation in organisational involvement, access to information, decision-making responsibilities, or professional autonomy, although these mechanisms were not directly assessed. The findings therefore support considering professional role when interpreting practice-environment assessments but do not indicate that professional category is a major determinant of those perceptions. Professional category also partly overlaps with management role and with professional experience, since nurse managers typically have longer careers and formal management responsibilities. To limit redundancy, role performed was not entered into the models, and apart from the strong collinearity between age and years of professional practice (r = 0.96; VIF ≈ 14), collinearity diagnostics did not indicate problematic multicollinearity; nevertheless, the independent contributions of category, role, and experience cannot be fully disentangled in the present data.

The individual-level multivariable models explained only 3.6–8.0% of the variance in PES-NWI dimensions. Rather than supporting a predominantly individual explanation of practice-environment perceptions, this limited explanatory capacity suggests that conventional sociodemographic and professional characteristics capture only a small part of the phenomenon. Variables more proximal to work organization, including workload, staffing patterns, organisational culture, leadership practices, autonomy, recognition, and career-development opportunities, may account for additional variability [36]. The organisation-level clustering identified in the multilevel models further supports the need to conceptualise the practice environment as an organisational as well as an individual-level phenomenon.

The exploratory profile analysis provides additional evidence that the uneven configuration observed at the aggregate level may reflect different combinations of practice-environment dimensions rather than a simple contrast between high- and low-scoring environments. More than one-third of participants (36.6%) were assigned to the strong foundations/staffing vulnerability profile (Profile 1), characterised by comparatively favourable care-quality foundations and nurse–physician relations alongside markedly weaker staffing and resource adequacy. This profile was observed across unfavourable, mixed, and favourable overall practice-environment classifications, suggesting that similar aggregate favourability levels may coexist with different multidimensional configurations. However, given the weak cluster separation (average silhouette width ≈ 0.21), the moderate-to-limited bootstrap stability (Jaccard coefficients 0.64–0.72), and the limited split-half reproducibility (median adjusted Rand index 0.31), the profiles should be understood as tendencies within a continuous multidimensional space rather than as robust or clearly distinct groups, and the 36.6% prevalence estimate should be regarded as imprecise (bootstrap 95% interval: 25.5–40.2%). These findings suggest that multidimensional, person-centred approaches may complement conventional PES-NWI summary classifications, although further validation in independent samples is necessary.

From an organisational perspective, the findings suggest that improvement strategies should not rely exclusively on aggregate practice-environment scores or interventions directed at individual professional competencies. Assessment and improvement efforts may benefit from simultaneously addressing participation in organisational governance, leadership support, professional recognition, and workforce adequacy, while recognising that these dimensions may not evolve uniformly. Longitudinal and intervention studies are required to determine whether modifying these organisational characteristics leads to sustained improvements in the practice environment and, ultimately, to better professional and patient outcomes.

### Limitations

This study has several limitations that should be considered when interpreting the findings. First, the cross-sectional design precludes establishing temporal ordering between variables and does not support causal inference. Accordingly, the observed associations between healthcare sector, professional characteristics, organisational participation, staffing adequacy, and the remaining PES-NWI dimensions should be interpreted as statistical relationships rather than evidence of causal effects or mechanisms. This limitation is particularly relevant to the exploratory indirect-effect analyses, which identify statistical decompositions of associations but cannot establish mediation in a causal sense.

A non-probability convenience sampling strategy was used at both the organisational and individual levels. The 29 participating healthcare organisations were not randomly selected from all Portuguese healthcare organisations, and participation by nurses was voluntary. Although the sample included 1004 nurses from different geographical regions and both healthcare sectors, the findings cannot be assumed to be representative of all nurses or healthcare organisations in Portugal. Organisations and nurses who agreed to participate may differ systematically from those who did not, introducing the possibility of selection bias.

No formal a priori sample-size calculation was performed because all eligible nurses accessible through participating organisations were invited to participate. In addition, the total number of nurses who received the survey invitation could not be determined precisely. Consequently, a response rate could not be calculated, and potential differences between respondents and non-respondents could not be examined. The magnitude and direction of any non-response bias therefore remain unknown.

Data were collected exclusively through a self-report electronic questionnaire. The findings reflect nurses’ perceptions of their practice environments and may have been influenced by individual expectations, professional experiences, organisational roles, and circumstances at the time of participation. Although the validated Portuguese version of the PES-NWI was used, the instrument does not directly measure objective staffing levels, workload, quality of care, patient safety, adverse events, or organisational performance. Accordingly, favourable or unfavourable PES-NWI scores should not be interpreted as direct evidence of actual clinical or organisational outcomes.

The electronic questionnaire required responses to all instrument items before submission, which prevented item-level missing data among completed questionnaires. However, this design may have contributed to survey abandonment among some potential participants. Because only fully submitted questionnaires were available for analysis, nurses who initiated but did not complete the questionnaire could not be characterised, and the extent to which their perceptions differed from those of completers is unknown.

The distribution of participants across healthcare sectors was markedly unbalanced. Of the 1004 nurses included, only 92 (9.2%) worked in the private sector, with 82 of these participants concentrated in two predominantly private organisations. This imbalance reduced the precision of private-sector estimates and limited the organisational diversity represented within the private sector. Consequently, estimated public–private differences may partly reflect characteristics of the specific organisations included in the study rather than systematic differences between healthcare sectors. Moreover, the non-probability sampling strategy limits the generalisability of these comparisons to the broader Portuguese healthcare system.

Although multilevel models accounted for the clustering of nurses within healthcare organisations, organisation-level estimates remain subject to uncertainty. The analyses included only 29 organisations with unequal cluster sizes, limiting the precision of variance-component estimates and the reliability of inference for organisation-related associations. The Kenward–Roger small-sample correction provided a sensitivity assessment of fixed-effect inference but could not overcome limitations related to the number and composition of participating organisations. Furthermore, leave-one-organisation-out analyses demonstrated that the statistical significance of several sector-related associations was sensitive to the exclusion of individual organisations, particularly one predominantly private organisation. These findings highlight the limited robustness of some sector-related estimates and reinforce the need for cautious interpretation. In addition, the dataset contained limited information on organisational characteristics, including governance arrangements, staffing policies, leadership models, organisational culture, professional autonomy, and career-development structures. Future studies should include a larger and more balanced sample of organisations, with sufficient representation from both healthcare sectors and more detailed organisational characteristics.

The study also did not include several objective characteristics of services and work organisation that may influence nurses’ perceptions of the practice environment. Nurse-to-patient ratios, workload, patient dependency and complexity, skill mix, unit type, staff-distribution models, and other operational indicators were not assessed. Their absence limits the ability to determine which specific organisational mechanisms underlie lower perceptions of staffing adequacy, participation, or leadership support and may partly explain the modest variance accounted for by the individual-level regression models.

The multidimensional profile analysis was exploratory and should be interpreted accordingly. The identified profiles represent data-driven configurations of relative PES-NWI dimensions within the present sample and should not be regarded as externally validated diagnostic categories or fixed organisational typologies. In particular, the labels applied to the three profiles (strong foundations/staffing vulnerability, relatively balanced, and resourced-but-unsupported) summarise their relative dimensional patterns and are interpretative rather than formally validated constructs. Internal stability was moderate to limited (mean bootstrap Jaccard coefficients 0.64–0.72; median split-half adjusted Rand index 0.31), and average silhouette widths of approximately 0.2 indicated weak separation between clusters; the profiles should therefore be understood as tendencies within a continuous multidimensional space rather than as sharply delimited groups, and replication in independent samples is required before these profiles can be considered generalisable.

The study included nurses from heterogeneous care settings and geographical areas, but the available analyses did not fully distinguish between hospitals, primary healthcare, specific clinical services, or other organisational contexts. Aggregating these settings may conceal important contextual differences in practice environments. This consideration is also relevant because the original PES-NWI was developed primarily in hospital contexts and may not capture all organisational characteristics equally across different models of care.

Multiple PES-NWI outcomes, group comparisons, post hoc tests, regression models, and exploratory indirect-effect analyses were examined without formal multiplicity adjustment in the primary analyses. This increases the risk of false-positive findings, particularly for isolated associations with *p*-values close to the conventional significance threshold. Although selected findings remained statistically significant under a Bonferroni-adjusted threshold of 0.01 within specified families of five outcome-specific tests, others did not, including the multilevel healthcare-sector associations for Nurse Manager Ability, Leadership and Support (D3) and Staffing and Resource Adequacy (D4). Furthermore, these sensitivity analyses did not account for all comparisons performed across the study. Consequently, findings should be interpreted with emphasis on effect estimates, confidence intervals, consistency across analyses, and substantive relevance rather than statistical significance alone.

Finally, data were collected between January 2023 and March 2023. The findings therefore reflect organisational conditions perceived during that period. Subsequent changes in workforce policies, healthcare-system organisation, leadership structures, staffing levels, or working conditions may limit the direct applicability of the estimates to the current Portuguese healthcare context. Nevertheless, the multidimensional and organisation-level patterns identified remain relevant for understanding how structural, professional, and relational characteristics may coexist within nursing practice environments and for generating hypotheses for longitudinal and intervention research.

## 5. Conclusions

This multicentre study identified an uneven multidimensional pattern in Portuguese nurses’ perceptions of their practice environments. Relatively favourable perceptions of Nursing Foundations for Quality of Care and Collegial Nurse–Physician Relations coexisted with less favourable perceptions of Nurse Participation in Organisational Affairs, Nurse Manager Ability, Leadership and Support, and Staffing and Resource Adequacy.

Exploratory person-centred profile analysis suggested that aggregate favourability classifications may conceal different configurations of perceived organisational strengths and vulnerabilities. More than one-third of participating nurses (36.6%; bootstrap 95% interval: 25.5–40.2%) were assigned to a strong foundations/staffing vulnerability profile, characterised by relatively favourable care-quality foundations and nurse–physician relations alongside markedly lower staffing adequacy scores. This configuration was observed across different levels of overall practice-environment favourability. However, the weak separation, moderate-to-limited stability, and limited reproducibility of the identified profiles require cautious interpretation and independent replication.

The findings also underscore the relevance of organisational context. Differences between healthcare organisations accounted for 4.2–12.8% of the variance across PES-NWI dimensions in the null multilevel models, and accounting for organisational clustering changed the statistical inference for some healthcare-sector associations. Only the association between sector and Nurse Participation in Organisational Affairs was consistent across all sensitivity analyses; the remaining sector associations were organisation-sensitive. Given the substantial imbalance between sectors and the concentration of private-sector participants in a small number of organisations, these comparisons should not be generalised to Portuguese public and private healthcare organisations as a whole.

Exploratory cross-sectional indirect-effect analyses identified statistical associations involving Nurse Participation in Organisational Affairs and selected PES-NWI dimensions. These findings do not establish mediation or causal pathways but suggest that participatory governance deserves further investigation as a potentially relevant organisational characteristic.

Overall, the findings support complementing aggregate practice-environment assessments with multidimensional and organisation-sensitive approaches. Future longitudinal, multilevel, and intervention studies are needed to validate the identified profiles, clarify the relationships between participation, leadership, staffing adequacy, and other practice-environment dimensions, and determine whether changes in organisational conditions are associated with improvements in nurses’ professional experiences and patient-related outcomes.

## Figures and Tables

**Figure 3:**
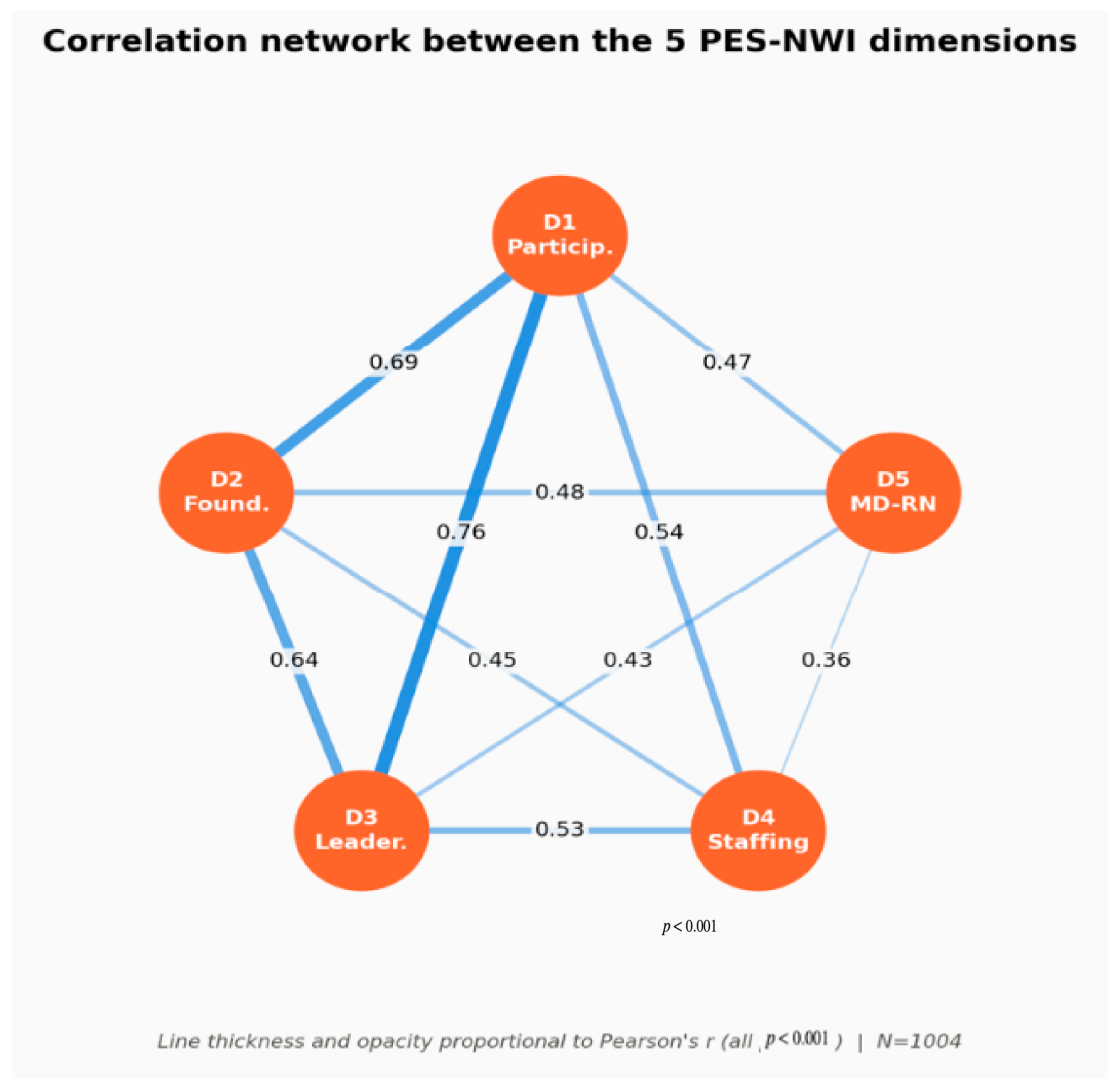
Network graph of Pearson correlations between the five PES-NWI dimensions (edge thickness and opacity proportional to r; all *p* < 0.001).

**Table 1:** Dimensional structure of the PES-NWI and distribution of items.

Dimensions	Items
D1 Nurse Participation in Organisational Affairs	5, 6, 11, 15, 17, 21, 23, 27, 28
D2 Nursing Foundations for Quality of Care	4, 14, 18, 19, 22, 25, 26, 29, 30, 31
D3 Nurse Manager Ability, Leadership and Support	3, 7, 10, 13, 20
D4 Staffing and Resource Adequacy	1, 8, 9, 12
D5 Collegial Nurse–Physician Relations	2, 16, 24

Legend: D1: Dimension 1; D2: Dimension 2; D3: Dimension 3; D4: Dimension 4; D5: Dimension 5.

**Table 2:** Internal consistency (Cronbach’s alpha) by dimension.

Dimension	Items	Cronbach’s Alpha
D1 Nurse Participation in Organisational Affairs	9	0.87
D2 Nursing Foundations for Quality of Care	10	0.84
D3 Nurse Manager Ability, Leadership and Support	5	0.83
D4 Staffing and Resource Adequacy	4	0.70
D5 Collegial Nurse–Physician Relations	3	0.83

**Table 3:** Classification of the practice environment according to the PES-NWI.

Result	Environment Type
1 or 0 dimensions > 2.5	Unfavourable Environment
2 or 3 dimensions > 2.5	Mixed Environment
4 or 5 dimensions > 2.5	Favourable Environment

**Table 4:** Sociodemographic and professional characterisation of participants (*N* = 1004).

Variable/Category	*N*	%
Sex		
Female	822	81.9
Male	182	18.1
Professional practice sector		
Public	912	90.8
Private	92	9.2
Regional Section of the Portuguese Nurses’ Association		
Centre	223	22.2
North	255	25.4
South	491	48.9
Madeira	34	3.4
Azores	1	0.1
Geographical area (district)		
Aveiro	81	8.1
Beja	33	3.3
Braga	41	4.1
Bragança	23	2.3
Coimbra	26	2.6
Évora	29	2.9
Faro	176	17.5
Guarda	35	3.5
Lisbon	68	6.8
Madeira	33	3.3
Portalegre	5	0.5
Porto	174	17.3
Santarém	97	9.7
Setúbal	103	10.3
Viana do Castelo	26	2.6
Viseu	54	5.4
Academic degree		
Bacharelato (pre-Bologna diploma)	11	1.1
Bachelor’s degree	690	68.7
Master’s degree	294	29.3
Doctoral degree	9	0.9
Postgraduate training		
Postgraduate diploma	186	18.5
Postgraduate nursing specialisation course	217	21.6
Both	268	26.7
Not applicable	333	33.2
Nursing specialty area		
Community Nursing	61	6.1
Community and Public Health Nursing	23	2.3
Medical-Surgical Nursing: Person in Critical Condition	128	12.7
Medical-Surgical Nursing: Person in Palliative Condition	10	1.0
Perioperative Nursing	23	2.3
Medical-Surgical Nursing: Person with Chronic Illness	16	1.6
Rehabilitation Nursing	111	11.1
Child Health and Paediatric Nursing	56	5.6
Maternal and Obstetric Health Nursing	58	5.8
Mental Health and Psychiatric Nursing	45	4.5
No specialist title	473	47.1
Professional category		
Generalist nurse	609	60.7
Specialist nurse	337	33.6
Nurse manager	58	5.8
Role performed		
Direct care provision	772	76.9
Management	68	6.8
Both	164	16.3
Employment status		
Open-ended Public Employment Contract (CTFP)	378	37.6
Open-ended Individual Labour Contract (CIT-TI)	568	56.6
Fixed-term Individual Labour Contract (CIT-TC)	24	2.4
Other	34	3.4

Note. CTFP = Public Employment Contract (Contrato de Trabalho em Funções Públicas); CIT = Individual Labour Contract (Contrato Individual de Trabalho).

**Table 5:** Descriptive statistics for continuous variables.

Variable	Min.	Max.	M	SD
Age (years)	22	67	42.58	9.76
Years of practice	0	44	19.44	9.97
Years at organisation	0	43	16.53	10.51
Distance to work (km)	0	83	11.97	12.17

Note. M = mean; SD = standard deviation; km = kilometres.

**Table 6:** PES-NWI dimension scores.

Dimension	Mean	SD
D1 Nurse Participation in Organisational Affairs	2.27	0.56
D2 Nursing Foundations for Quality of Care	2.78	0.51
D3 Nurse Manager Ability, Leadership and Support	2.37	0.66
D4 Staffing and Resource Adequacy	2.28	0.61
D5 Collegial Nurse–Physician Relations	2.70	0.61

**Table 7:** Sensitivity analysis by sector (Mann–Whitney U test).

Dimension	U	*p*
D1 Nurse Participation in Organisational Affairs	29,434	<0.001
D2 Nursing Foundations for Quality of Care	31,403	<0.001
D3 Nurse Manager Ability, Leadership and Support	32,518	<0.001
D4 Staffing and Resource Adequacy	33,814	0.002
D5 Collegial Nurse–Physician Relations	29,382	<0.001

**Table 8:** Sensitivity analysis by professional category (Kruskal–Wallis test).

Dimension	H	*p*
D1 Nurse Participation in Organisational Affairs	47.81	<0.001
D2 Nursing Foundations for Quality of Care	50.10	<0.001
D3 Nurse Manager Ability, Leadership and Support	48.50	<0.001
D4 Staffing and Resource Adequacy	18.89	<0.001
D5 Collegial Nurse–Physician Relations	11.62	0.003

**Table 9:** Comparison of PES-NWI subscales by practice sector.

Dimension	Public M (SD)	Private M (SD)	t	df	*p*	d
D1 Nurse Participation in Organisational Affairs	2.24 (0.55)	2.55 (0.60)	−5.07	1002	<0.001	−0.55
D2 Nursing Foundations for Quality of Care	2.76 (0.51)	2.99 (0.54)	−4.14	1002	<0.001	−0.45
D3 Nurse Manager Ability, Leadership and Support	2.34 (0.65)	2.59 (0.71)	−3.45	1002	<0.001	−0.38
D4 Staffing and Resource Adequacy	2.26 (0.61)	2.46 (0.60)	−3.03	1002	0.003	−0.33
D5 Collegial Nurse–Physician Relations	2.67 (0.60)	2.98 (0.60)	−4.61	1002	<0.001	−0.50

Note. d = Cohen’s d; df = degrees of freedom. Negative values of t and d indicate higher mean scores in the private-sector group.

**Table 10:** ANOVA of PES-NWI subscales by professional category.

Dimension	Generalist M (SD)	Specialist M (SD)	Manager M (SD)	F	*p*	η^2^
D1 Nurse Participation in Organisational Affairs	2.22 (0.54)	2.28 (0.56)	2.78 (0.57)	28.02	<0.001	0.053
D2 Nursing Foundations for Quality of Care	2.74 (0.49)	2.78 (0.55)	3.21 (0.38)	23.55	<0.001	0.045
D3 Nurse Manager Ability, Leadership and Support	2.32 (0.65)	2.35 (0.66)	2.95 (0.54)	25.29	<0.001	0.048
D4 Staffing and Resource Adequacy	2.23 (0.61)	2.32 (0.60)	2.57 (0.55)	9.89	<0.001	0.019
D5 Collegial Nurse–Physician Relations	2.67 (0.61)	2.72 (0.62)	2.93 (0.56)	5.34	0.005	0.011

Note. η^2^ = eta squared; M = mean; SD = standard deviation. Post hoc comparisons (Games–Howell): Manager > Generalist and Manager > Specialist for D1–D5 (adjusted *p* ≤ 0.030); Specialist vs. Generalist not statistically significant for any dimension (adjusted *p* ≥ 0.072).

**Table 12:** (**a**) Comparison of candidate k-means solutions based on ipsatised PES-NWI scores (N = 1004). (**b**) Individual-level profiles identified from ipsatised PES-NWI scores (N = 1004). (**c**) Absolute PES-NWI subscale scores, mean ipsatised deviations, and individual-level practice-environment favourability across the three exploratory profiles.

(**a**)					
**k**	**WCSS**	**Average Silhouette Width**	**Calinski–Harabasz Index**	**Davies–Bouldin Index**	**Smallest Cluster, n (%)**
2	508.6	0.219	316.1	1.634	485 (48.3%)
3	416.3	0.212	303.8	1.395	264 (26.3%)
4	364.1	0.196	279.2	1.412	220 (21.9%)
5	327.9	0.191	259.8	1.342	131 (13.0%)
6	305.1	0.191	238.1	1.348	123 (12.3%)
(**b**)					
**Profile**		**N (%)**	**Highest-Scoring Dimension**	**Lowest-Scoring Dimension**	**Within-Profile Range**
Profile 1: Strong foundations/staffing vulnerability		367 (36.6)	D5 (3.03)	D4 (1.93)	1.10
Profile 2: Relatively balanced		373 (37.2)	D2 (2.91)	D4 (2.34)	0.57
Profile 3: Resourced-but-unsupported		264 (26.3)	D4 (2.68)	D1 (2.07)	0.61
(**c**)					
**Characteristic**			**Profile 1: Strong Foundations/Staffing Vulnerability (n = 367)**	**Profile 2: Relatively Balanced (n = 373)**	**Profile 3: Resourced-but-Unsupported (n = 264)**
D1 Nurse Participation in Organisational Affairs			2.17 (−0.25)	2.52 (−0.06)	2.07 (−0.35)
D2 Nursing Foundations for Quality of Care			2.76 (+0.34)	2.91 (+0.33)	2.62 (+0.20)
D3 Nurse Manager Ability, Leadership and Support			2.22 (−0.20)	2.71 (+0.13)	2.08 (−0.34)
D4 Staffing and Resource Adequacy			1.93 (−0.49)	2.34 (−0.24)	2.68 (+0.26)
D5 Collegial Nurse–Physician Relations			3.03 (+0.61)	2.42 (−0.16)	2.64 (+0.22)
Mean of the five dimensions			2.42	2.58	2.42
Individual classification: unfavourable, n (%)			107 (29.2%)	123 (33.0%)	92 (34.8%)
Individual classification: mixed, n (%)			192 (52.3%)	90 (24.1%)	107 (40.5%)
Individual classification: favourable, n (%)			68 (18.5%)	160 (42.9%)	65 (24.6%)

(**a**) Note. WCSS = within-cluster sum of squares. Higher silhouette and Calinski–Harabasz values and lower Davies–Bouldin values indicate better cluster separation. (**b**) Note. Profiles were identified using k-means clustering of ipsatised PES-NWI subscale scores. Values shown for the highest- and lowest-scoring dimensions represent absolute mean subscale scores within each profile, rather than ipsatised deviations. Within-profile range represents the difference between the highest and lowest absolute mean subscale scores. Profile labels are descriptive and interpretative and should not be considered externally validated categories of nursing practice environments. D1 = Nurse Participation in Organisational Affairs; D2 = Nursing Foundations for Quality of Care; D3 = Nurse Manager Ability, Leadership and Support; D4 = Staffing and Resource Adequacy; D5 = Collegial Nurse–Physician Relations. (**c**) Note. Values for the five PES-NWI dimensions are presented as absolute mean subscale scores, with mean ipsatised deviations in parentheses. Ipsatised deviations were calculated by subtracting each participant’s mean score across the five PES-NWI dimensions from their respective subscale scores. Positive and negative deviations indicate dimensions rated relatively above or below the participant’s overall mean, respectively, and should not be interpreted as indicators of absolute practice-environment favourability. Profiles were identified using ipsatised scores and represent exploratory configurations rather than validated categories. Individual-level favourability was classified separately according to Lake’s criterion applied to each participant’s absolute subscale scores. Percentages for favourability classifications are calculated within each profile.

**Table 13:** Intraclass correlation coefficients (ICCs) and comparison of standard errors for the practice-sector effect (OLS vs. multilevel models).

Dimension	ICC (Null Model)	OLS SE (Sector)	Multilevel SE (Sector)	SE Ratio (Multilevel/OLS)
D1 Nurse Participation in Organisational Affairs	5.9%	0.061	0.075	1.23
D2 Nursing Foundations for Quality of Care	12.8%	0.056	0.102	1.81
D3 Nurse Manager Ability, Leadership and Support	9.4%	0.073	0.117	1.60
D4 Staffing and Resource Adequacy	4.2%	0.068	0.090	1.32
D5 Collegial Nurse–Physician Relations	5.4%	0.068	0.093	1.36

Note. ICC = variance attributable to healthcare organisation/total variance, from a random-intercept-only model.

**Table 14:** (**a**) Multilevel model estimates (random intercept by healthcare organisation): private vs. public sector (reference: public). (**b**) Consistency of healthcare-sector associations (private vs. public) across analytical approaches.

(**a**)						
**Dimension**		**B (Multilevel)**	**95% CI**	***p***	**N Organisations**	**N Nurses**
D1 Nurse Participation in Organisational Affairs		0.323	[0.168, 0.478]	<0.001	29	1004
D2 Nursing Foundations for Quality of Care		0.170	[−0.033, 0.374]	0.100	29	1004
D3 Nurse Manager Ability, Leadership and Support		0.243	[0.005, 0.481]	0.046	29	1004
D4 Staffing/Resource Adequacy		0.208	[0.025, 0.391]	0.027	29	1004
D5 Collegial Nurse–Physician Relations		0.307	[0.119, 0.495]	0.002	29	1004
(**b**)						
**Dimension**	**OLS (** Table 11 **c–g)**	**Multilevel (Satterthwaite; Table 14a)**	**Multilevel (Kenward–Roger)**	**Bonferroni (α = 0.01), MULTILEVEL**	**Leave-One-Organisation-Out (29 Iterations)**	**Classification**
D1 Nurse Participation in Organisational Affairs	*p* < 0.001	*p* < 0.001	*p* < 0.001	Significant	Significant in all 29	Consistent
D2 Nursing Foundations for Quality of Care	*p* < 0.001	*p* = 0.100	*p* = 0.107	Not significant	Non-significant in 26	Organisation-sensitive
D3 Nurse Manager Ability, Leadership and Support	*p* < 0.001	*p* = 0.046	*p* = 0.048	Not significant	Non-significant in 14	Organisation-sensitive
D4 Staffing and Resource Adequacy	*p* = 0.001	*p* = 0.027	*p* = 0.030	Not significant	Non-significant after excluding one predominantly private organisation (*p* = 0.175)	Organisation-sensitive
D5 Collegial Nurse–Physician Relations	*p* < 0.001	*p* = 0.002	*p* = 0.003	Significant	Non-significant after excluding one predominantly private organisation (*p* = 0.121)	Organisation-sensitive

(**a**) Note. B = unstandardised regression coefficient; CI = confidence interval. Models included a random intercept for healthcare organisation and were adjusted for sex, age, professional category, years of professional practice, and employment status. Healthcare sector was coded as 0 = public and 1 = private, with the public sector as the reference category. Models were estimated using restricted maximum likelihood (REML). Reported estimates correspond to the primary multilevel models; Kenward–Roger sensitivity results are presented separately in the text. Confidence intervals and *p*-values were obtained from t-distributions with denominator degrees of freedom approximated using the Satterthwaite method, as described in Section 2.5. (**b**) Note. OLS = ordinary least squares. “Consistent” = statistically significant in all analytical approaches; “organisation-sensitive” = not statistically significant in at least one approach. All sector coefficients were positive (higher scores in the private sector) in all models and iterations. Classifications refer to the present sample and do not imply generalisable public–private differences.

**Table 15:** Exploratory indirect-effect analysis of associations between healthcare sector and selected PES-NWI dimensions through nurse participation and staffing and resource adequacy.

Pathway	Direct Effect (b, *p*)	Indirect Effect (b)	Total Association (c)	Indirect/Total (%)	Individual Bootstrap 95% CI	Cluster Bootstrap 95% CI
Sector → D1 → D2	0.037, *p* = 0.370	0.194	0.231	84.0	[0.113, 0.275]	[0.059, 0.273]
Sector → D1 → D5	0.151, *p* = 0.011	0.154	0.305	50.5	[0.089, 0.223]	[0.049, 0.213]
Sector → D4 → D2	0.155, *p* = 0.002	0.075	0.230	32.6	[0.025, 0.127]	[0.020, 0.180]
Sector → D4 → D5	0.234, *p* < 0.001	0.071	0.305	23.3	[0.024, 0.121]	[0.018, 0.173]

Note. D1 = Nurse Participation in Organisational Affairs; D2 = Nursing Foundations for Quality of Care; D4 = Staffing and Resource Adequacy; D5 = Collegial Nurse–Physician Relations. In each pathway, the first dimension represents the intervening variable and the second represents the outcome. Coefficients are unstandardised; healthcare sector was coded as 0 = public and 1 = private. Individual bootstrap confidence intervals were based on 3000 resamples, whereas cluster-bootstrap confidence intervals were based on 5000 resamples of healthcare organisations. No additional covariates were included in the component models. These exploratory cross-sectional statistical decompositions do not establish causal mediation. Total association (c) = direct component (c′) + indirect component (a × b); the proportion represented by the indirect component was calculated as (a × b/c) × 100 and is reported descriptively; it does not represent the proportion of a causal effect explained.

## Data Availability

The data supporting the findings of this study are not publicly available due to privacy and ethical considerations related to the protection of participants’ confidentiality. Data may be made available by the corresponding author upon reasonable request, subject to applicable ethical and data protection requirements.
